# Prevalence of Sickle Cell Disease Among Anaemic Children Attending Mbeya Referral Hospital in Southern Tanzania

**DOI:** 10.24248/EAHRJ-D-18-00015

**Published:** 2018-11-23

**Authors:** Augustine M Musyoka, Kavavila Zebedayo, Blandina T Mmbaga

**Affiliations:** a Kilimanjaro Christian Medical University College, Moshi, Tanzania; b Kilimanjaro Clinical Research Institute, Moshi, Tanzania; c Mbeya Referral Hospital, Mbeya, Tanzania; d Kilimanjaro Christian Medical Centre, Moshi, Tanzania

## Abstract

**Background:**

Sickle cell disease (SCD) is a common genetic haematological disorder present in most countries in sub-Saharan Africa. In Tanzania, between 50% and 75% of the children born with SCD die before reaching the age of 5 years. The objective of this study was to determine the prevalence of SCD in children under 5 years of age attending Mbeya Referral Hospital between March and April 2014.

**Methods:**

We conducted a hospital-based, cross-sectional, descriptive study in which 50 children under 5 were included at Mbeya Referral Hospital in southern Tanzania. Full blood counts were conducted using SYSMEX KX 21 and SYSMEX XT 2000i haematology analysers. The presence of haemoglobin S was determined using the sodium metabisulfite sickling test on blood samples with haemoglobin levels less than 10 g/dl.

**Results:**

Blood samples from 50 infants and children under 5 were tested for sickle cell anaemia. Of these, 9 (18%) participants were found to be sickling test positive, 5 (55.6%) of whom were male and 4 (44.4%) were female. Almost half (n=4, 44.4%) of the SCD-positive children were between 25 and 36 months old, while the rest were between 13 and 24 months (n=2, 22.2%), 37 and 48 months (n=1, 11.1%), and 49 and 60 months (n=2, 22.2%) of age.

**Conclusion:**

At our facility, among children under 5 with serum haemoglobin levels <10 g/dl, the prevalence of SCD was 18%. This might pose a substantial public health challenge in the region. More and larger studies are needed to help map out the sickle cell burden throughout the country to guide policy and management strategies.

## INTRODUCTION

Sickle cell disease (SCD) is the most common hereditary condition that affects the structure of haemoglobin. The abnormal sickle-shaped structure that is characteristic of SCD occurs as a result of a person inheriting either 1 (heterozygous AS) or 2 (homozygous SS) sickle cells genes from their parents. Haemoglobin SS (HbSS) homozygosity, the most severe form of sickle cell anaemia, leads to a structural variation of the haemoglobin globin chains, particularly beta (*β*)-globin chains, making them polymerise upon deoxygenation, which can lead to vaso-occlusion in the microcirculation and subsequent ischaemia, pain, and tissue damage.^1^

SCD poses considerable public health challenges,^2,3^ particularly in sub-Saharan Africa – a region also characterised by a high burden of malaria.^4,5^ Worldwide, over 300,000 children are born with a sickled haemoglobin (HbS) disorder each year.^6^ Sub-Saharan Africa has the greatest burden of SCD, with more than 70% of the 300,000 global cases occurring in the region.^7–9^ Projected estimates indicate that the number of newborns with SCD will exceed 400,000 by 2050.^5^

In HbS, the structural *β*-globin polypeptide gene experiences a substitution mutation on the codon for the sixth amino acid (GAG to GTG), which leads to an amino acid substitution of valine for glutamic acid at position 6 of the 146-amino-acid polypeptide chain.^3^ This causes crystallisation and polymerisation of the abnormal HbS as a result of deoxygenation during normal oxygen transport processes.^10^ The red blood cells carrying this abnormal haemoglobin gradually become sickle-shaped and are thus unable to pass through microcapillaries. The trapped cells cause vaso-occlusion, which in turn leads to a repeated cycle of ischaemia and reperfusion.^11^ Sickle cells are also susceptible to mechanical damage, which can lead to chronic haemolytic anaemia, and recurrent crises can cause end-organ damage.^12^

### Distribution of SCD in Africa

The sickle cell trait and malaria exhibit a similar geographic distribution, owing to the evolutionary link between the 2 entities. Sickle cell trait is known to protect against the development of severe *Plasmodium falciparum* malaria, which explains the high frequency of the sickle cell gene in African regions most affected by *P. falciparum* malaria.^8^ The protective benefits of the sickle cell trait are most consequential during early childhood, because this is a crucial development period, and it is when humans are most at risk of mortality from severe malaria. Sickle cell carriers are more likely to escape childhood mortality from malaria and, therefore, subsequently pass the abnormal haemoglobin gene to their offspring. Although a single abnormal gene is protective against *P. falciparum* malaria, the inheritance of 2 abnormal genes leads to SCD and does not confer such protection.^4,8^ Additionally, many of the complications of SCD are severe and life-threatening, and many individuals with the disease die before reaching reproductive age.^9^

### Public Health Impact of Sickle Cell Disease in Children

SCD accounts for about 5% of deaths among African children under 5, including more than 9% of under-5 deaths in West Africa and up to 16% in individual West African countries.^9^

Makani and colleagues, in 2011, reported 5.7% mortality among children with SCD in Dar es Salaam, Tanzania. The factors that were strongly associated with death among these children included low haemoglobin levels and high total and conjugated bilirubin levels, with the highest incidence of death being reported among children under 5.^7^

Treating SCD involves therapy with hydroxyurea, a ribonucleotide reductase inhibitor, which is given as a daily oral dose to prevent the acute or chronic complications of the disease.^14^ Despite this, SCD remains difficult to treat, with the only potential cure being haematopoietic stem cell transplantation, the use of which is limited by its high cost and difficulties with human leukocyte antigen compatibility.^15^

Administration of prophylactic antibiotics to all infants diagnosed with SCD is recommended, as these children are more likely to suffer from bacterial infections, especially those caused by *Streptococcus pneumoniae*.^16^

Weatherall reported that individuals carrying the HbAS genotype are protected against malaria and that severe malaria is more likely to occur in homozygous (HbSS) individuals as well as in normal healthy people (HbAA).^17^ Malaria infection increases the risk of ischaemic crises and childhood morbidity and mortality.^18,19^ In the deoxygenated condition, HbS has poor solubility, forming polymers in red cells leading to changes in the red cell membrane and metabolism, causing the cells to become rigid and distorted with a sickle shape. The sickled cells haemolyse easily, adhere to vascular endothelium and one another, block small blood vessels, and become sequestered in the spleen.^20^

Regionally, in 2010, an estimated 79% of newborns with SCD were in sub-Saharan Africa, and this proportion is expected to increase to 88% by 2050.^5^ Muoneke et al reported that SCD and malaria were significantly associated with severe anaemia among children under 5 in South East Nigeria.^21^

In Tanzania, the frequency of HbAS is estimated to be around 13%, and there are about 8,000 annual births of homozygous HbSS children, compared to 302 in Jamaica and 1,500 in the United States, for example.^7^ It is also estimated that between 50% and 75% of the 8,000 Tanzanian children born with SCD die before the age of 5 years. In 2013, Simbauranga et al reported a 21% SCD prevalence among paediatric patients with anaemia in Mwanza, Tanzania. Severe paediatric anaemia was associated with SCD in this study, wherein 34 (11%) of 309 patients showed homozygous inheritance (HbSS) of SCD, while 31 (10%) patients were heterozygous (HbAS).^22^

Therefore, in this study, we sought to investigate the prevalence of SCD among children below 5 attending Mbeya Referral Hospital (MRH) in the Southern Highlands Zone of Tanzania.

## METHODS

### Study Design and Setting

We conducted a hospital-based, cross-sectional study at MRH, which is a 477-bed tertiary health-care facility that has been operating as the referral centre for the southern part of Tanzania since 1985. MRH serves a catchment population of over 6 million, and it has extensive infectious disease medical clinics, inpatient services, training facilities, and a referral clinical laboratory. MRH covers the regions of Ruvuma, Rukwa, Iringa, Njombe, Katavi, and Mbeya.

### Participant Criteria

Children under 5 who presented with haemoglobin levels less than 10 g/dl were eligible and screened for SCD.

### Data Collection

We collected data for this study over 2 months, starting in March 2014 and targeted children whose physician-requested laboratory investigations included a full blood count or haemoglobin determination. We did not inspect the clinical data because we only aimed to estimate the prevalence of SCD among children with low haemoglobin levels. Once the full blood count was done, we looked at children with low haemoglobin levels (less than 10.0 g/dl) and classified them, based on local population-validated reference ranges, as having moderate (7 to 9.99 g/dl) or severe (<7 g/dl) anaemia.

We obtained parental written informed consent before collecting venous blood specimens, by standard venepuncture procedures, into a 2 ml K_3_ or K_2_ EDTA tubes, which were sent to the MRH clinical laboratory. Thin blood films were prepared from EDTA-anticoagulated venous blood and stained with 5% Giemsa for morphological examination. For patients found to be anaemic or showing a red cell distribution width greater than 20%, a peripheral smear evaluation was done followed by a sickle cell test. Full blood counts were determined using the SYSMEX KX 21 and SYSMEX XT 2000i haematology analysers (Sysmex Corporation, Japan). Sickling tests were performed using the 2% sodium metabisulphite-based haemoglobin deoxygenation method. EDTA-anticoagulated blood was placed on a slide and mixed with 2% sodium metabisulphite, covered with a cover glass, and then incubated at room temperature for 20 minutes and examined for sickling. All positive results were reported at the first examination, and the negative samples were re-examined once per hour for 3 hours then incubated overnight and examined the following day if still negative.

### Quality Control and Quality Assurance

All laboratory investigations were done at the MRH laboratory, which is accredited by the Southern African Development Community Accreditation System. The hospital operates under high quality control standards. All reagents were checked for expiry dates and reconstituted according to the manufacturer's instructions. Daily maintenance checks were conducted to ensure proper functioning of the instruments. Quality control runs for haematological analysers were done daily before running patient samples using tri-level control (low, normal, and high). Known sickle cell-positive and negative samples were used as controls for the sickling tests.

### Ethical Consideration

We obtained permission to conduct this study from the MRH administration and the regional medical officer, through Tumaini University, Makumira Kilimanjaro Christian Medical University College, Office of the Dean, in the Faculty of Medicine. The guardian or caretaker of each participant provided informed consent. Participation was voluntary and did not affect the care provided to the patients.

### Data Analysis

A single data collector entered the data, which were checked for accuracy by another individual. The analysis was done using Statistical Software for Social Sciences (SPSS), version 20 (IBM Corp., Armonk, NY, USA). Descriptive statistics (means, medians, standard deviations, and proportions) were estimated.

## RESULTS

### Sociodemographic Characteristics

We enrolled 50 children, 25 females and 25 males, with ages ranging from 1 to 60 months and a mean age (± standard deviation [SD]) of 22.82 ± 18 months (**Table 1**).

**TABLE 1.** Age Categories and Sex Distribution of Study Participants (N=50)

Age (Months)	Males n (%)	Females n (%)	Total n (%)
1–12	9 (18)	11 (22)	20 (40)
13–24	7 (14)	5 (10)	12 (24)
25–36	5 (10)	3 (6)	8 (16)
37–48	3 (6)	2 (4)	5 (10)
49–60	1 (2)	4 (8)	5 (10)
**Total**	**25 (50)**	**25 (50)**	**50 (100)**

### Prevalence of Sickle-Cell Anaemia by Age and Sex

We tested 50 blood samples for SCD, among which 9 (18%) were positive for SCD. Of the 9 children found to be sickle cell-positive, 5 (55.6%) were males and 4 (44.4%) were females. The age and gender distributions of the children with SCD are displayed in **Table 2**.

**TABLE 2.** Sickle Cell Test Results by Age and sex Categories of the Study Participants (N=50)

	Sickling Test		
Age (Months)	Positive n (%)	Negative n (%)	Total n (%)
1–12	Male: 0 (0)	12 (24)	20 (40)
	Female: 0 (0)	8 (16)	
13–24	Male: 2 (4)	6 (12)	12 (24)
	Female: 0 (0)	4 (8)	
25–36	Male: 2 (4)	2 (4)	8 (16)
	Female: 2 (4)	2 (4)	
37–48	Male: 0 (0)	3 (6)	5 (10)
	Female: 1 (2)	1 (2)	
49–60	Male: 1 (2)	2 (4)	5 (10)
	Female: 1 (2)	1 (2)	
**Total**	**Male: 5 (10)**	**25 (50)**	**50 (100)**
	**Female: 4 (8)**	**16 (32)**	

### Haemoglobin Levels

Forty-two (84%) children had haemoglobin levels between 6.0 and 9.9 g/dl; the rest had haemoglobin levels below 6.0 g/dl (**Table 3**). Four (44.4%) of the 9 children with SCD had haemoglobin levels between 6.0 and 9.9 g/dl, and 5 (55.6%) had haemoglobin levels below 6.0 g/dl. The mean haemoglobin level for children with SCD was 6.4 g/dl, compared with 8.1 g/dl among children who had a negative sickle cell test result.

**TABLE 3.** Haemoglobin Levels by Age Category (N=50)

	Haemoglobin Level		
Age (Months)	0-5.9 g/dl n (%)	6.0-9.9 g/dl n (%)	Total n (%)
1–12	4 (8)	16 (32)	20 (40)
13–24	2 (4)	10 (20)	12 (24)
25–36	0 (0)	8 (16)	8 (16)
37–48	0 (0)	5 (10)	5 (10)
49–60	2 (4)	3 (6)	5 (10)
**Total**	**8 (16)**	**42 (84)**	**50 (100)**

## DISCUSSION

This cross-sectional study aimed to estimate the prevalence of SCD in southern Tanzania. We report a high prevalence (18%) of SCD among anaemic children under 5 in this population, which is similar to prevalence reports from eastern Uganda and Qatif, Saudi Arabia, of 17.5% and 17.9%, respectively.^23,24^ However, the prevalence observed in our study was higher than the 3% reported for sub-Saharan Africa.^25^ The prevalence reported in this study may have been higher because of the small sample size and the selection of only children with low haemoglobin values. However, this prevalence may not deviate substantially from reality, based on the occurrence of sickle cells recorded in routine patient care laboratory tests.

SCD positivity was most frequently detected among children aged between 25 and 36 months in this study. Haemoglobin levels below 5 g/dl were most frequently encountered in children between 1 and 12 months of age; surprisingly, none of these children tested positive for SCD. Many of the severely anaemic children may have had iron-deficiency anaemia, malaria, or other causes of haemolysis common among children. This would imply that low haemoglobin should not necessarily be associated with sickle cell anaemia in this population. We also might have missed some children with SCD as a result of our choice to target low haemoglobin levels as the starting point of our SCD screening.

In this study, the prevalence of SCD among males was slightly higher than the prevalence among females, with a ratio of 1.25:1. Similarly, a study conducted in a rural hospital in central India reported a slightly higher prevalence among males (1.07:1),^26^ compared to findings in Saudi Arabia, where SCD was reported to occur at a ratio of 1:1 between males and females.^27^ The preselection of low haemoglobin levels could have been the cause of this slight variation.

The mean haemoglobin level among sickle cell-positive children was 6.5 g/dl, which was similar to what was reported from a cohort of children with SCD at the Red Cross Children's Hospital in Cape Town, South Africa.^28^ In our study, 34% of children were severely anaemic (haemoglobin <7 g/dl), which is a slightly lower prevalence of severe anaemia than the 39% reported from a community-based study conducted in southeastern Tanzania.^29^ This difference might be because children in southeastern Tanzania are more at risk of acute malaria and, therefore, have higher rates of severe anaemia. The risk of mortality is high with low haemoglobin levels, as previously reported by a surveil-lance study in Tanzania.^7^

### Limitations

The study period was short, limiting the study to the few patients who visited the hospital during the study period. Likewise, resource limitations, particularly those related to funding the student who carried out this study as part of his bachelor's degree in health laboratory sciences, may have affected our sample size. It is possible that we failed to detect SCD in some patients because we did not use haemoglobin electrophoresis in this study. Despite these limitations, we managed to recruit children with SCD, which was a target of the study.

## CONCLUSION

Our study found that the prevalence of SCD in children under 5 in the southern highlands of Tanzania was high at 18%. Larger studies may help to map out the sickle-cell burden among the various regions of the country, which, in turn, will help better inform the planning of management and control strategies. Future studies are needed to determine if newborn screening and early identification may complement early preventive measures to improve quality of life.
